# ctDNA-based detection of residual disease: implications for management of non-metastatic NSCLC

**DOI:** 10.3389/fonc.2026.1951454

**Published:** 2026-09-04

**Authors:** Charline Leclercq, Mylène Wespiser, Gaëlle Tachon, Adrien Buisson, Maurice Pérol, Aurélie Swalduz

**Affiliations:** 1 Department of Medical Oncology, Centre Léon Bérard, Lyon, France; 2 Univ Lyon, Claude Bernard Lyon 1 University, Institut national de la santé et de la recherche médicale (INSERM) 1052, Centre national de la recherche scientifique (CNRS) 5286, Centre Léon Bérard, Cancer Research Center of Lyon, Lyon, France; 3 Department of Biopathology, Centre Léon Bérard, Lyon, France

**Keywords:** circulating tumor DNA, early-stage lung cancer, liquid biopsy, minimal residual disease, non-small cell lung cancer, risk stratification

## Abstract

Despite advancements in perioperative and consolidative immunotherapy in early-stage to locally advanced non-small cell lung cancer (NSCLC), recurrence rates remain substantial. In this context, liquid biopsy has transformed the oncological landscape by enabling the non-invasive detection of tumor-derived components, including DNA, cells, and proteins, from biological fluids. Although plasma-based circulating tumor DNA (ctDNA) analysis is routinely used in metastatic NSCLC to identify actionable genomic alterations at diagnosis and mechanisms of acquired resistance to targeted therapies, its application in non-metastatic disease represents a pivotal challenge. Leveraging minimal residual disease (MRD) detection through ctDNA offers the potential for early risk stratification, enabling the identification of patients who may benefit from treatment escalation, such as intensified adjuvant or consolidative regimens, while sparing low-risk patients from unnecessary toxicity through de-escalation strategies. This review summarizes the biological and technological foundations underlying ctDNA-based MRD detection, including tumor-informed and non-tumor-informed approaches, and examines the current evidence supporting its clinical utility in non-metastatic NSCLC. We also highlight key technical and clinical challenges and discuss future perspectives to optimize integration of ctDNA MRD assessment into routine clinical practice.

## 1 Introduction

While the utility of liquid biopsy across all stages of non-small cell lung cancer (NSCLC) continues to expand, its clinical implementation remains largely restricted to the metastatic setting. Current ESMO and ASCO guidelines recommend plasma-based genotyping primarily for the identification of resistance mechanisms following progression on targeted therapies or when tissue acquisition is infeasible or insufficient for molecular testing (1, 2). However, circulating tumor DNA (ctDNA) analysis holds significant promise for early detection and the identification of molecular residual disease (MRD) following curative-intent treatment of localized NSCLC, which could ultimately be the source of a later clinical recurrence.

Over the last decade, the therapeutic landscape for localized NSCLC has been profoundly transformed by the introduction of perioperative immunotherapy-based strategies and adjuvant targeted therapies for oncogene-driven disease. Perioperative chemoimmunotherapy regimens have demonstrated significant improvements in event-free survival and, more recently, overall survival, while adjuvant targeted therapies have established new standards of care in *EGFR*-mutated (ADAURA), *ALK*-(ALINA), and *RET-*(LIBRETTO-432) rearranged NSCLC (3–9). Despite these advances, the residual risk of relapse remains substantial (10). Reported 2-year recurrence rates remain approximately 36% following neoadjuvant (3) or perioperative chemoimmunotherapy approaches (11, 12), while 3-year recurrence rates of approximately 15% and 22% have been reported in patients receiving adjuvant osimertinib (6) and alectinib, respectively (7).

ctDNA-based MRD detection has been incorporated as an exploratory endpoint in virtually all major perioperative NSCLC trials and has emerged as a promising biomarker for risk stratification. Beyond its prognostic value, it holds considerable potential to guide personalized therapeutic strategies, through both escalation strategies for high-risk patients, and de-escalation strategies for those achieving molecular clearance. However, several analytical and clinical issues must be overcome before its integration into routine practice. This review summarizes the biological basis of ctDNA detection, discusses available MRD assays, and highlights the current clinical evidence and challenges that must be overcome before routine clinical implementation can be achieved.

## 2 Biological bases

### 2.1 General principles

Liquid biopsy involves the isolation and analysis of circulating tumor cells (CTCs) or tumor-derived analytes, such as tumor-associated proteins, extracellular vesicles, and ctDNA, from biological fluids (13). To date, ctDNA remains the only clinically validated analyte, while others continue to undergo investigation. Total cell-free DNA (cfDNA) comprises all plasma extracellular DNA, of which ctDNA is the tumor-derived fraction, typically very small (<0.01–10%) (2, 14). Its short half-life (8 minutes to 2.5 hours) makes it a near-real-time surrogate for tumor burden (15, 16). As with all liquid biopsy approaches, sensitivity and specificity remain the major challenges. MRD detection requires extreme analytical sensitivity, as postoperative residual tumor burden yields ctDNA fractions orders of magnitude lower than in advanced disease, frequently at or below 0.01–0.1% (17). Specificity can be compromised by clonal hematopoiesis of indeterminate potential (CHIP) which refers to the acquisition of somatic mutations in hematopoietic stem cells, leading to clonal expansion: because most cfDNA originates from hematopoietic cells, CHIP-associated variants may be misclassified as tumor-derived (18, 19). To mitigate this issue, paired sequencing of patient-derived leukocytes is increasingly used; however, removing the contribution of other sources of background genetic alterations may be challenging (20). To address these challenges, ctDNA assays are broadly classified into two categories: tumor-informed and tumor-agnostic (also referred to as tumor-naive) approaches.

### 2.2 Tumor-informed assays

Tumor-informed assays first sequence the patient’s tumor to identify somatic alterations that can subsequently be tracked in plasma. By focusing on patient-specific variants, these assays maximize the signal-to-noise ratio and reduce the risk of detecting non-tumor-derived alterations. Several tumor-informed assays have been developed. Signatera, one of the most extensively studied, uses whole-exome sequencing (WES) of tumor and matched germline DNA to identify patient-specific clonal mutations (19, 21, 22). More recently, technological advances have focused on improving sensitivity at ultra-low ctDNA levels. PhasED-Seq detects co-localized (phased) mutations on the same DNA fragment, sharply reducing noise and false positives (23, 24), while whole-genome sequencing (WGS)-based approaches (e.g., MRDetect, NeXT Personal) expand the number of trackable variants (25, 26) (**Table 1**). In NSCLC, NeXT Personal showed clinical sensitivities of 61.5% (landmark) and 86% (longitudinal), with specificities above 90% (27). The main limitations of tumor-informed approaches include long turnaround times, the need for adequate tumor tissue, which can be problematic in cases of inaccessible biopsies or pathological complete response (pCR), and their inability to capture genomic alterations that emerge during treatment (18).

**Table 1:** Selected ctDNA assays for MRD detection in NSCLC.

Tumor-informed assays					
Assay	Technique	Number of genes	LoD (%VAF)	Trial (pts evaluable/included, n); study design	Key clinical data
AMP patient-specific cfDNA enrichment panels	Detection of SNV; WES-based	196	0.003%	TRACERx (39) (131/197); Prospective observational cohort, NSCLC treated with surgery	**ctDNA status is a prognostic factor (post-operative):** – OS for ctDNA+ vs ctDNA− pts: HR 5.3 (p = 1 × 10^-9^) (landmark) – FFR for ctDNA+ vs ctDNA− pts: HR 6.8 (p = 6 × 10^-13^) (landmark) **Longitudinal ctDNA monitoring predicts relapse:** – 20% of landmark-negative pts became ctDNA+ during follow-up; biological relapse preceded imaging in many pts
Signatera	Detection of SNV; WES-based	16	0.01%	IMpower010 (22) (600/1005); Phase III, surgery and adjuvant CT followed by atezolizumab vs BSC	**ctDNA status is a prognostic factor (adjuvant setting):** – DFS for post-CT ctDNA− vs ctDNA+ pts: 31.3 vs 4.2 months (atezolizumab) and 13.3 vs 3.9 months (BSC)
Invitae/ArcherDX Personalized Cancer Monitoring (PCM)^§^	Detection of SNV; WES-based	18-50	0.008%	CheckMate 816 (43) (66/358); Phase III, IB-IIIA (7^th^ TNM) neoadjuvant CT+nivolumab vs CT followed by surgery	**ctDNA clearance correlates with pathological response (depth):** – ctDNA clearance observed in >90% of pts reaching pCR vs 20% in pts with >50% residual viable tumor
Invitae/ArcherDX Personalized Cancer Monitoring (PCM)^§^	Detection of SNV; WES-based	18-50	0.008%	CheckMate 77T (46) (140/461); Phase III, IIA-IIIB (8^th^ TNM) neoadjuvant CT+nivolumab and adjuvant nivolumab vs neoadjuvant CT+placebo and adjuvant placebo	**pCR is a stronger prognostic factor than ctDNA (neoadjuvant setting):** – Nivolumab arm — without pCR, ctDNA clearance vs no clearance was not significantly associated with improved EFS (slight trend, HR 0.70) – When ctDNA cleared, absence of pCR was associated with worse outcome: EFS for ctDNA−/pCR vs ctDNA−/no pCR: HR 0.29 **ctDNA clearance correlates with pathological response:** – >98% of pts with persistent pre-surgical ctDNA failed to achieve pCR
Invitae/ArcherDX Personalized Cancer Monitoring (PCM)^§^	Detection of SNV; WES-based	18-50	0.008%	AEGEAN (44, 45) (186/802); Phase III, IIA-IIIB (8^th^ TNM) neoadjuvant CT+durvalumab and adjuvant durvalumab vs neoadjuvant CT+placebo and adjuvant placebo	**ctDNA status is a prognostic factor (post-operative):** – 12-month DFS: 89.3% (95% CI 82.6–93.5) in ctDNA− vs 14.3% (95% CI 2.4–36.3) in ctDNA+ pts **ctDNA clearance does not necessarily imply pCR:** – PPV of ctDNA clearance for pCR (durvalumab arm): 49% at C2D1; 40% at pre-surgery **ctDNA clearance correlates with pathological response:** – 100% of pts with pCR and >93% with MPR had ctDNA clearance by neoadjuvant C4D1 **ctDNA clearance is associated with better outcomes (durvalumab arm):** – At C2 — EFS: HR 0.30 (95% CI 0.12–0.71); OS: HR 0.32 (95% CI 0.14–0.72) – At C4 — EFS: HR 0.26 (95% CI 0.13–0.54) **In patients not reaching pCR, ctDNA status is a prognostic factor:** – ctDNA clearance vs no clearance significantly associated with improved EFS (HR 0.35)
NeXT Personal	WGS	Up to 1800	0.000167%	NeoADAURA (48) (189/358); Phase III, IIA-IIIB (8^th^ TNM) *EGFR*+ neoadjuvant osimertinib+CT vs osimertinib vs placebo	**ctDNA clearance correlates with pathological response:** – MPR rates for neoadjuvant ctDNA clearance vs no clearance: 24% vs 6%
NeXT Personal	WGS	Up to 1800	0.000167%	LAURA (49) (52/216); Phase III, unresectable stage III *EGFR*+ CRT followed by consolidative osimertinib vs placebo	**ctDNA status is a prognostic factor (post-CRT):** – post-CRT ctDNA+: 63% clinical sensitivity, 86% specificity for PFS events – MRD detection preceded clinical relapse in 79% of pts, median lead time 5.1 months
RaDaR	Detection of SNV; WES-based	48	0.001%	ADAURA (47) (202/682); Phase III, IB-IIIA (7^th^ TNM) *EGFR*+ adjuvant osimertinib vs placebo	**ctDNA status is a prognostic factor (adjuvant setting):** – Pre-osimertinib (postoperative and post-CT) ctDNA+ pts had shorter DFS than ctDNA− pts – Among 18 pre-osimertinib ctDNA+ pts, 17 relapsed – Among pre-osimertinib ctDNA− pts, 17/98 relapsed (osimertinib arm; mostly after discontinuation, n=12/17) and 51/85 (placebo arm) – ctDNA detection preceded radiological recurrence by a median of 4.7 months (sensitivity 65%, specificity 95%)
PhasED-Seq	Detection of «phased-variants»; WES-based	Up to 622	0.000094%	–	–
MRDetect	WGS	–	0.001%	–	–
Tumor-agnostic assays					
Assay	Technique	Number of genes	LoD (%VAF)	Trial (pts evaluable/included, n); study design	Key clinical data
cSMART	Multiplexed inverse PCR	9 (*EGFR, KRAS, ERBB2, PIK3CA, BRAF, MET, TP53, ALK, RET*)	0.01%	DYNAMIC, 2019 (38) (25/36); prospective observational cohort, NSCLC treated with surgery	**ctDNA status is a prognostic factor (post-operative):** – RFS for ctDNA+ vs ctDNA− at D3 and D30: 278 vs 637 days (p = 0.02) – OS for ctDNA+ vs ctDNA− at D3 and/or D30: 434 vs 720 days (p = 0.018)
CAPP-Seq	Hybridization-based	139	0.02%	Moding et al., 2020 (63) (53/65); prospective observational cohort, NSCLC treated with CRT ± IO	**ctDNA status is a prognostic factor (post-CRT):** – Undetectable ctDNA after CRT → low progression: IO group 0% at 12 months; non-IO group 25–30% – IO group — 12-month FFP: 0% (ctDNA+) vs 87.5% (ctDNA−) (p < 0.0001, HR 84.4, 95% CI 12.3–579.9) – FFP similar between IO and non-IO groups (NS; limited sample; p = 0.23)
iDES-enhanced CAPP-Seq	Detection of duplex variants	128	0.002%	Chaudhuri et al., 2017 (37) (37/40); prospective observational cohort, NSCLC treated with surgery or CRT	**ctDNA status is a prognostic factor (post-operative or post-CRT):** – 36-month PFS for ctDNA+ vs ctDNA−: 0% vs 93% (p < 0.001, HR 43.4) – 36-month OS for ctDNA+ vs ctDNA−: 20% vs >80% (p < 0.001, HR 10.9) – MRD detection preceded clinical relapse in 72% of pts, median lead time 5.2 months
Oncomine Pan-Cancer Cell-Free Assay	Amplicon-based	52	–	NADIM (42) (40/46); Phase II single arm, IIIA (7^th^ TNM) neoadjuvant CT+nivolumab, surgery followed by adjuvant nivolumab	**ctDNA status is a prognostic factor (post-neoadjuvant):** – ctDNA clearance after neoadjuvant treatment → better OS vs no clearance (HR 6.88, 95% CI 1.29–36.78; p = 0.007) – 5-year OS for clearance vs no clearance: 92.3% vs 59.2% – 5-year PFS for clearance vs no clearance: 85.2% vs 60.6%

Analytical characteristics and key clinical findings of representative tumor-informed and tumor-agnostic assays.

AMP, anchored-multiplex PCR; BSC, best supportive care; CRT, chemoradiotherapy; CT, chemotherapy; DFS, disease-free survival; EFS, event-free survival; *EGFR*+, *EGFR*-mutated; FFP, freedom from progression; FFR, freedom from recurrence; IO, immunotherapy; LoD, limit of detection; MPR, major pathological response; MRD, molecular residual disease; OS, overall survival; pts, patients; pCR, pathological complete response; PFS, progression-free survival; PPV, positive predictive value; RFS, relapse-free survival; SNV, single-nucleotide variant; VAF, variant allelic frequency; WES, whole-exome sequencing; WGS, whole-genome sequencing;.

^§^ArcherDX PCM and Invitae PCM denote the same tumor-informed assay (ArcherDX was acquired by Invitae in 2020).

Bold text highlights the key findings supported by the corresponding results presented underneath.

### 2.3 Tumor-agnostic assays

Unlike tumor-informed assays, tumor-agnostic assays do not require prior characterization of the primary tumor. Rather than tracking patient-specific mutations, they interrogate plasma directly for molecular features suggestive of residual disease, which, depending on the platform, may include recurrent somatic mutations, fragmentomic profiles, epigenetic signatures, or integrated multi-analyte signatures (28) (**Table 1**). Beyond mutation-based assays, emerging tumor-agnostic approaches increasingly exploit orthogonal biological signals such as cfDNA fragmentation patterns and DNA methylation profiles to further improve MRD detection (29, 30). Fragmentomic leverages the shorter, aberrantly fragmented nature of ctDNA. Whereas physiologic cfDNA is nucleosome-protected (peak at 167 bp), ctDNA shows a truncated peak near 143 bp, allowing tumor-derived DNA to be identified independently of specific alterations (30–32). In parallel, epigenetic approaches, particularly DNA methylation profiling, exploit tumor-specific patterns that arise early in oncogenesis and persist throughout tumor evolution (31). These approaches may provide highly stable biomarkers that often precede the appearance of somatic mutations, potentially offering superior sensitivity than tumor-agnostic mutation-based panels for ctDNA detection (33). Tumor-agnostic assays offer shorter turnaround, lower complexity, and applicability when tissue is insufficient or after pCR. However, lacking patient-specific variants, they are generally less sensitive and more susceptible to biological noise particularly associated with CHIP. This reduced specificity may lead to a false diagnosis of relapse and, consequently, to unnecessary treatment, exposing patients to potentially adverse effects and additional treatment burden.

### 2.4 Comparison

The choice between these approaches reflects a trade-off between analytical performance and operational feasibility. Direct comparison between assays remains challenging because of substantial heterogeneity in study designs, patient populations, sampling schedules, and assay characteristics.

Nevertheless, current evidence generally favors tumor-informed approaches for MRD detection. In a meta-analysis by Lu et al. encompassing 30 studies and 3,287 patients undergoing NSCLC resection, tumor-informed assays achieved higher specificity than tumor-agnostic approaches, both in landmark analyses (97% vs. 93%) and longitudinal surveillance (96% vs. 88%). Differences in sensitivity were comparatively modest (44% vs. 42% for landmark; 79% vs. 76% for longitudinal) (34). Interpretation of these findings requires caution, however. Crucially, the meta-analysis was dominated by East Asian cohorts, known for a higher prevalence of *EGFR* mutations, which are systematically included in most predefined tumor-agnostic panels. This may have artificially enhanced the performance of tumor-agnostic assays in this dataset, suggesting that the sensitivity benefit of tumor-informed approaches could be even larger in Caucasian populations. Beyond this meta-analysis, a recent single-institution study provided a direct head-to-head comparison of tumor-naïve, tumor-informed fixed, and tumor-informed personalized panels within the same resected NSCLC cohort. It showed similar results, with post-surgical landmark sensitivity increasing across these strategies (13.6%, 27.3%, and 40.9%, respectively) and higher specificity for both tumor-informed assays; MRD positivity by the personalized assay was strongly associated with shorter disease-free survival (35). Importantly, the tumor-agnostic assays evaluated in these comparisons rely exclusively on mutation-based gene panels. Increasingly, multi-modal assays, with fragmentomic and/or methylation-based approaches, are being evaluated to optimize MRD detection accuracy (36). These next-generation approaches aim to combine the analytical performance of tumor-informed strategies with the operational advantages of tumor-agnostic workflows and may ultimately represent the future direction of MRD detection in resectable NSCLC. However, the question of which assay should be used in this setting is, to date, far from resolved.

## 3 Clinical evidence and applications

### 3.1 Clinical evidence of ctDNA-based MRD across clinical settings in retrospective cohorts

Early evidence for the clinical validity of ctDNA-based MRD in NSCLC came largely from retrospective analyses and small cohorts that paved the way for the biomarker sub-studies embedded within prospective trials discussed below. In 2017, Chaudhuri et al. first showed that post-treatment ctDNA detection, whether at a landmark or during longitudinal follow-up, predicted inferior progression-free survival (PFS) and overall survival (OS), and preceded radiographic progression by a median of 5.2 months (37). The 2019 DYNAMIC study similarly linked postoperative ctDNA detection to worse OS (38), and TRACERx showed that longitudinal monitoring identified recurrence in 20% of patients not yet detectable by imaging (39). Across cohorts, molecular relapse has consistently preceded radiological relapse by a median of 3.5–7 months (40, 41). Beyond the binary MRD call, higher postoperative ctDNA burden also carries prognostic weight after adjustment for histology and stage (27).

### 3.2 Clinical evidence of ctDNA-based MRD across clinical settings in randomized trials

#### 3.2.1 Peri-operative immunotherapy strategies

ctDNA has emerged as a powerful biomarker within perioperative strategies for resectable NSCLC. In IMpower010, postoperative MRD negativity predicted improved disease-free survival (DFS), whereas persistent ctDNA identified a subgroup at high risk of relapse (22). In NADIM, baseline ctDNA levels were associated with PFS and OS, outperforming radiological criteria for prognostic discrimination (42). In CheckMate 816, patients achieving pCR after neoadjuvant chemo-immunotherapy or chemotherapy had markedly higher 2-year event-free survival (EFS) than those without pCR, and both ctDNA clearance and the depth of pathological response correlated with outcome, although pathological assessment outperformed ctDNA clearance and radiological response in predicting EFS (43). Consistent findings emerged from the phase III AEGEAN trial of perioperative durvalumab: ctDNA clearance during neoadjuvant treatment was strongly associated with pCR and major pathological response (MPR). However, 50–60% of patients who cleared ctDNA still failed to reach pCR, showing that ctDNA clearance does not equate to the absence of residual disease (44). In a post-surgical landmark analysis, 12-month DFS was markedly lower among patients with detectable versus undetectable MRD in both arms (45). In CheckMate 77T, among patients without pCR, ctDNA clearance was associated with substantially better EFS than ctDNA persistence (46). In summary, postoperative ctDNA status is a consistently validated prognostic biomarker, and longitudinal ctDNA assessment enables early prediction of relapse. Although ctDNA clearance correlates with pathological response, it should not be considered equivalent to pCR, which retains greater prognostic value than ctDNA in the neoadjuvant context (**Table 1**).

#### 3.2.2 Targeted therapies adjuvant strategies

Comparable observations have been reported in oncogene-addicted NSCLC. In an exploratory analysis of ADAURA, postoperative MRD-positive patients had shorter DFS but still derived substantial benefit from adjuvant osimertinib versus placebo, even though all eventually relapsed (47). In NeoADAURA, neoadjuvant osimertinib (with or without chemotherapy) achieved higher ctDNA clearance rates than chemotherapy alone, and clearance was strongly associated with higher MPR rates (48). Overall, ctDNA status is likewise prognostic in the adjuvant setting, while in the neoadjuvant setting clearance correlates with pathological response (**Table 1**).

#### 3.2.3 Consolidative strategies after chemo-radiotherapy

Unlike more recent studies, the pivotal PACIFIC trial, which established consolidation durvalumab after chemoradiotherapy (CRT), did not incorporate MRD assessment. In LAURA (**Table 1**), MRD was also prognostic in this setting, and MRD-based detection of progression preceded radiological progression by a median of ~5 months (49). Notably, the PFS benefit from osimertinib was observed irrespective of post-CRT MRD status, suggesting that, at least with current assays and in this small exploratory subset, MRD status alone is not yet sufficient to guide treatment individualization here.

Taken together, these data establish ctDNA-based MRD as one of the strongest prognostic biomarkers currently available in localized NSCLC. Nevertheless, its limited sensitivity makes it an imperfect surrogate for residual disease, as a proportion of ctDNA-negative patients still relapse, and its ability to guide therapeutic decision remains to be demonstrated.

### 3.3 Potential therapeutic implications

The most promising application of ctDNA-based MRD lies in guiding treatment individualization. Perioperative decisions currently rely on clinicopathological factors (tumor size, nodal status, stage) that only indirectly estimate residual disease and poorly capture the biological heterogeneity of resectable NSCLC. Moreover, while neoadjuvant chemoimmunotherapy is well established, the specific contribution of the adjuvant immunotherapy component remains uncertain: no randomized trial has directly compared neoadjuvant chemoimmunotherapy alone with a full perioperative approach (4, 5, 50, 51), a gap the ongoing ADOPT-Lung trial (NCT06284317) is going to address (52). MRD-guided strategies are therefore attractive, since prolonged adjuvant immunotherapy may be unnecessary for all patients, whereas persistent MRD could identify those more likely to benefit from alternative approaches (novel immunotherapies, antibody-drug conjugates, targeted therapies, or other emerging agents).

**Figure 1:**
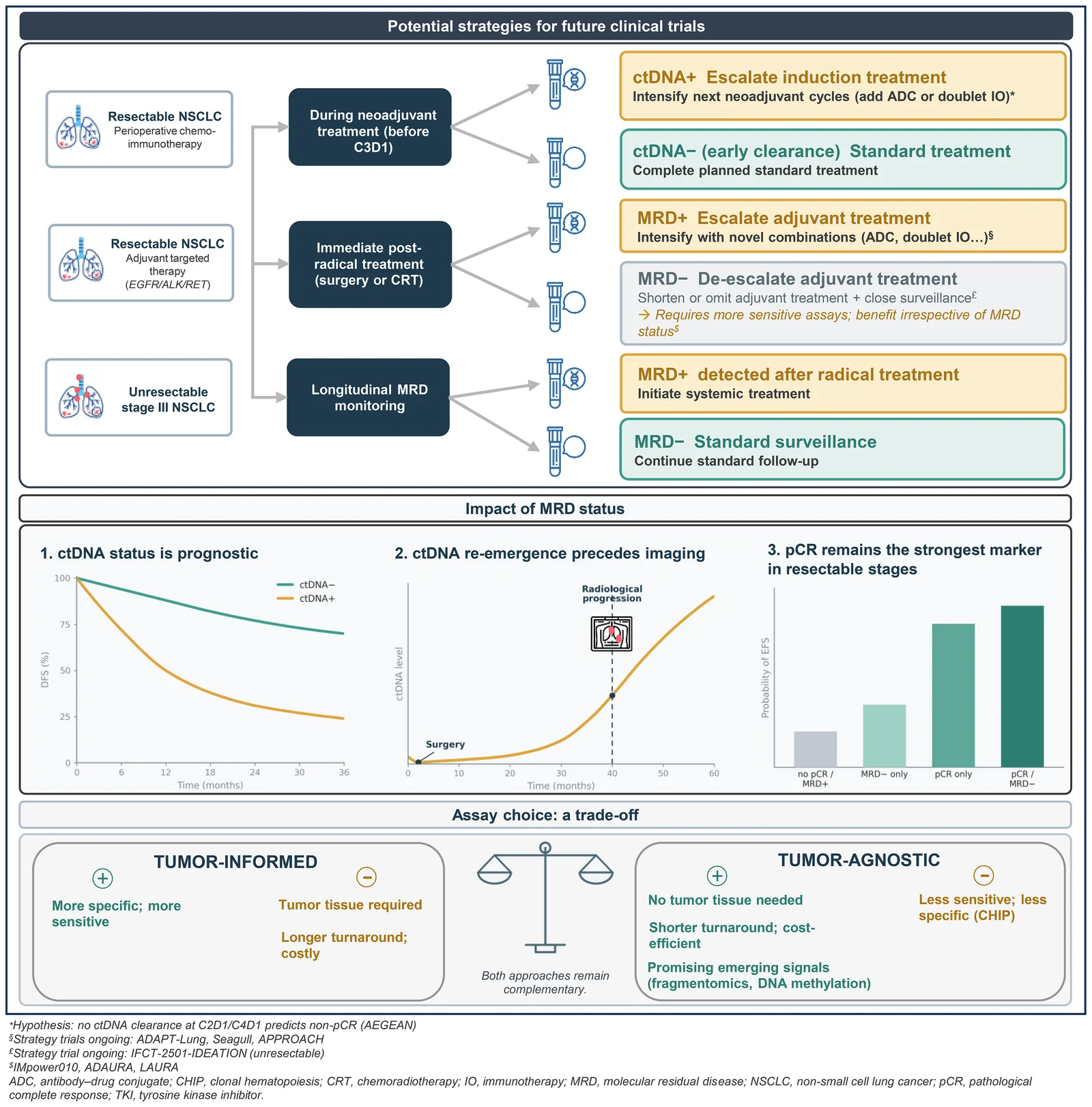
A ctDNA/MRD-guided decision investigational-framework across non-metastatic NSCLC settings. Proposed, investigational integration of ctDNA-based MRD across 3 curative-intent settings (perioperative chemo-immunotherapy, adjuvant targeted therapy, and consolidation after chemoradiotherapy). MRD positivity/ctDNA persistence (yellow) flags candidates for escalation and MRD negativity/ctDNA clearance (green) for standard or de-escalated management, with relevant strategy trials indicated. None of these strategies has yet been evaluated in clinical trials, and their efficacy remains to be established.The second panel illustrates 3 cross-cutting concepts. The last panel summarizes the trade-off between tumor-informed and tumor-agnostic assays.

One potential application is treatment escalation in high-risk patients (**Figure 1**). Some patients relapse despite low-stage tumors, suggesting an aggressive biological profile undetected by conventional staging. ctDNA detection is strongly associated with recurrence risk (19, 53), and could thus identify high-risk patients who would otherwise not be selected for adjuvant therapy. The APPROACH trial is investigating patients with stage III *EGFR*-mutated NSCLC who receive neoadjuvant aumolertinib before surgery or CRT. After local treatment, patients in the standard arm receive adjuvant aumolertinib. In the experimental arm, treatment is guided by MRD status: MRD-positive patients receive aumolertinib, while MRD-negative patients are observed without treatment. Aumolertinib is restarted if a patient later switches from an MRD-negative to an MRD-positive status during follow-up (54). ctDNA dynamics during neoadjuvant treatment may also justify escalation: the absence of ctDNA clearance enables early identification of patients unlikely to achieve pCR, as shown in AEGEAN (44), so on-treatment ctDNA monitoring could be used to intensify neoadjuvant therapy with the aim of improving pCR rates (55).

Conversely, ctDNA may also support treatment de-escalation (**Figure 1**). Because a substantial proportion of patients do not relapse after surgery, MRD assessment could identify them for treatment de-escalation, reducing toxicity, improving quality of life, and limiting costs. However, the sensitivity of current assays is probably still too low to support de-escalation. In IMpower010, a DFS and OS benefit was seen with atezolizumab regardless of ctDNA status; in ADAURA, 37% (68/183) of MRD-negative patients relapsed, and adjuvant osimertinib benefited the overall cohort (22, 47). In LAURA, both MRD-positive and MRD-negative patients benefited from consolidative osimertinib after CRT (49). Dedicated strategy trials are underway. ADAPT-Lung (NCT07120698) is a phase II trial enrolling stage II–IIIB NSCLC without pCR after neoadjuvant chemo-immunotherapy, and evaluating adjuvant sintilimab for MRD-positive versus surveillance for MRD-negative patients (56). The phase II Seagull trial (NCT05286957) compares a control arm based on adjuvant chemo-immunotherapy regardless of MRD status with an MRD-guided arm (adjuvant chemo-immunotherapy if MRD-positive, chemotherapy alone if MRD-negative) (57). IFCT-2501-IDEATION will randomize unresectable stage III NSCLC without progression after CRT between standard durvalumab consolidation and MRD-guided consolidation.

The clinical value of ctDNA may extend beyond a binary MRD call: longitudinal monitoring captures kinetics (clearance, persistence, and re-emergence), providing with a more dynamic picture of treatment efficacy and recurrence risk than a single postoperative landmark measurement.

## 4 Discussion and future directions

Despite its considerable promise, several challenges preclude the routine implementation of ctDNA-guided management in non-metastatic NSCLC.

First, residual ctDNA burden may fall below current detection thresholds, underscoring the need for greater analytical sensitivity. Second, standardization is lacking across sequencing platforms, genomic targets, bioinformatic pipelines, positivity thresholds, and sampling schedules; as a result, reported sensitivities and MRD positivity rates vary widely, which hampers direct comparisons between studies. The optimal timing and frequency of testing also remain uncertain, though longitudinal monitoring appears more sensitive than landmark analyses (34, 58).

Practical barriers also persist. Tumor-informed assays in particular require adequate tissue, personalized panel design, prolonged turnaround, and substantial cost. These upfront costs might be offset by better patient selection (avoiding unnecessary adjuvant therapy and its toxicities or detecting recurrence earlier). In England, in suspected stage III or IV lung cancers, an economic evaluation of ctDNA use at diagnosis involving 1,296 patients estimated annual savings of £11 million (59). Metastatic and localized NSCLC are, however, associated with substantially different therapeutic strategies and costs. Therefore, cost-effectiveness analyses specifically focusing on non-metastatic NSCLC are warranted.

Ultimately, the field has largely established the prognostic value of ctDNA-based MRD in localized NSCLC, but the key unanswered question is whether ctDNA-guided interventions improve patients’ outcomes. Consequently, prospective trials stratifying treatment by MRD status are essential to establish clinical utility and justify routine implementation. In other solid tumors, such trials have already begun to reshape practice. In stage II colon cancer, the phase II DYNAMIC trial first showed that a ctDNA-guided strategy could safely de-escalate adjuvant treatment, halving chemotherapy use (15% vs. 28%) without compromising 2-year recurrence-free survival (93.5% vs. 92.4%) (60). Its phase 2/3 successor DYNAMIC-III in stage III disease confirmed ctDNA as a strong prognostic classifier (3-year RFS 14% vs. 79% by post-treatment ctDNA status) but tempered expectations: escalation in ctDNA-positive patients did not improve outcomes, and de-escalation narrowly failed to meet noninferiority (61). In muscle-invasive bladder cancer, the single-arm phase II TOMBOLA trial used serial ctDNA after neoadjuvant chemotherapy and cystectomy to start early atezolizumab at molecular relapse (median lead time 90 days); 60% of ctDNA-positive patients treated at MRD positivity achieved a complete response, while ctDNA-negative patients were safely spared immediate immunotherapy (62). Collectively, these cross-organ trials provide a template for the randomized, MRD-stratified interventional trials now required in non-metastatic NSCLC.

MRD assessment may also enrich clinical trials: as MRD-positivity frequently precedes radiographic relapse, ctDNA has the potential to serve as an early surrogate endpoint for evaluating novel strategies (next-generation immunotherapies, antibody-drug conjugates, cancer vaccines, combinations), increasing event rates, reducing sample-size requirements, and accelerating perioperative drug development.

In conclusion, while ctDNA-based MRD detection in non-metastatic NSCLC is a potent predictor of relapse, its clinical utility remains constrained by insufficient sensitivity, a lack of standardization, and the absence of evidence that earlier detection improves outcomes. Notably, tumor-agnostic assays are inherently more applicable in routine practice, as they require no tumor tissue, yet the evidence accumulated so far rests almost exclusively on their mutation-based versions, whose sensitivity remains insufficient. Combining these with orthogonal tumor-agnostic signals, such as fragmentomic and methylation profiles, may therefore be the most promising route to improve sensitivity while preserving applicability, an approach still largely underexplored in this setting. Ultimately, harmonized assays and carefully designed, MRD-stratified interventional trials will be essential for MRD to move beyond a prognostic marker and enter clinical guidelines in localized NSCLC.
